# IL-6 promotes drug resistance through formation of polyploid giant cancer cells and stromal fibroblast reprogramming

**DOI:** 10.1038/s41389-021-00349-4

**Published:** 2021-09-29

**Authors:** Na Niu, Jun Yao, Robert C. Bast, Anil K. Sood, Jinsong Liu

**Affiliations:** 1 Departments of Anatomic Pathology, The University of Texas MD Anderson Cancer Center, Houston, Texas 77030 USA; 2 Departments of Molecular and Cellular Oncology, The University of Texas MD Anderson Cancer Center, Houston, Texas 77030 USA; 3 Departments of Experimental Therapeutics, The University of Texas MD Anderson Cancer Center, Houston, Texas 77030 USA; 4 Departments of Gynecologic Oncology and Reproductive Medicine, The University of Texas MD Anderson Cancer Center, Houston, Texas 77030 USA

**Keywords:** Cancer microenvironment, Tumour immunology

## Abstract

To understand the role of polyploid giant cancer cells (PGCCs) in drug resistance and disease relapse, we examined the mRNA expression profile of PGCCs following treatment with paclitaxel in ovarian cancer cells. An acute activation of IL-6 dominated senescence-associated secretory phenotype lasted 2–3 weeks and declined during the termination phase of polyploidy. IL-6 activates embryonic stemness during the initiation of PGCCs and can reprogram normal fibroblasts into cancer-associated fibroblasts (CAFs) via increased collagen synthesis, activation of VEGF expression, and enrichment of CAFs and the GPR77 + /CD10 + fibroblast subpopulation. Blocking the IL-6 feedback loop with tocilizumab or apigenin prevented PGCC formation, attenuated embryonic stemness and the CAF phenotype, and inhibited tumor growth in a patient-derived xenograft high-grade serous ovarian carcinoma model. Thus, IL-6 derived by PGCCs is capable of reprogramming both cancer and stromal cells and contributes to the evolution and remodeling of cancer. Targeting IL-6 in PGCCs may represent a novel approach to combating drug resistance.

## Introduction

Most deaths from ovarian cancer occur as a result of treatment resistance and relapse following chemotherapy [1–3]. Polyploid giant cancer cells (PGCCs), which can be either mononucleated or multinucleated with variable atypical nuclear morphology, are commonly observed in high-grade serous ovarian carcinoma (HGSC), particularly the following chemotherapy. Remarkably, little attention has been paid to these cells in the cancer research community because they were traditionally thought to be nonviable owing to their senescent nature and inability to execute mitosis [4].

In contrast to this widely held belief, we and others have recently provided evidence that PGCCs are indeed viable and can grow progressively by amitotic budding, splitting, and bursts of proliferation from mononucleated or multinucleated giant cells [5–12]. PGCCs formed in response to therapeutic stress can acquire embryonic stemness and generate new drug-resistant daughter cells [6, 7]. Moreover, single PGCCs are capable of initiating metastasis or tumor growth in immunosuppressed nu/nu mice [11–13]. Thus, PGCCs can activate embryonic program for dedifferentiation [6, 14] and represent a default cellular and developmental mechanism that occurs in response to therapy stress to initiate reprogramming for drug-resistant cells [4, 15–17].

Tumor microenvironment and inflammation is thought to be critical for cancer initiation, development, and progression and acquisition of drug resistance [18–21]. Inflammatory molecules such as IL-6, IL-8, IL-1β, and TNF-α have been shown to contribute to senescence and cancer initiation and progression in multiple cancers including breast and colorectal cancers [22, 23], and IL-6 is a therapeutic target in ovarian cancer [24, 25]. Stress-induced IL-6- and IL-8-activated fibroblasts provide fertile soil for tumor seeds to progress and evolve [26]. After chemotherapy, PGCCs are abundantly increased in radiation or post-chemotherapy or treated cells and cancer tissues [5–9], although the underlying mechanisms for how PGCCs communicate with the microenvironment remain unclear.

We have previously shown that the formation of PGCCs can facilitate the drug resistance following the treatment of paclitaxel [6, 7]. In the current report, we examined the mRNA expression profile in the initiation stage of PGCC formation and studied the mechanisms involved in embryonic stemness activation and the paracrine signals that mediate communication with the stromal fibroblasts.

## Materials and Methods

### Cell lines and treatment

Two human ovarian cancer cell lines, Hey and SKOV3; and patient-derived xenograft (PDX) cell line (MDACC-HGSC-1, derived from human HGSC) were cultured in a corresponding medium as described previously [6, 7]. The origin and purity of all cell lines were verified with short tandem repeat (STR) analysis before their use in MD Anderson’s Characterized Cell Line Core (https://www.mdanderson.org/education-and-research/resources-for-professionals/scientific-resources/core-facilities-and-services/characterized-cell-line-core-facility/index.html).

Paclitaxel (500 nM, Sigma), recombinant human IL-6 protein (R&D Systems, Minneapolis, MN, USA), IL-6R alpha antibody (R&D Systems, for in vitro use only), IL-6R alpha antibody (tocilizumab, for in vivo use in PDX models; Genentech, San Francisco, CA, USA), and apigenin (Sigma) were used in test groups as described in detail below.

### Apoptosis and senescence analysis

To detect the apoptosis and senescence of PGCCs at recovery day 7, Annexin V: FITC Apoptosis Detection kit II (BD), and β-Gal staining were performed as described previously [7].

### Cell labeling and spheroid formation

To visualize the morphology of nuclei, Hey and SKOV3 cells were labeled with pBabe-H2B-GFP as described previously [7]. At recovery day 7, PGCCs from testing groups were dissociated with trypsin (Sigma), then 5000 PGCCs were cultured in 3 ml human iPS cell medium for another 7 days with low-attachment dishes as reference [6]. The number of spheroids with diameter > =200 µm were calculated in test groups as described [27].

### RNA sequencing and gene ontology analysis

Total RNA was extracted from PGCCs treated with paclitaxel only (at recovery day 3) and budded daughter cells of Hey and SKOV3 as described previously [6]. Library construction and deep sequencing analysis (50 M reads/sample, DNB-Seq 150PE) were performed in the MD Anderson Science Park Next-Generation Sequencing Facility on an Illumina HiSeq 1000 sequencing system (Illumina Inc., San Diego, CA, USA). Clean fastq files were mapped to the human transcriptome (hg38) using salmon v0.14.1 to generate counts and TPM for each transcript. The summed mapped counts for all samples ranged from 29.6 to 30.6 million reads. All RNA-seq data is publicly accessible in Gene Expression Omnibus (GEO) (https://www.ncbi.nlm.nih.gov/geo/query/acc.cgi?acc=GSE178745)

### Verification with quantitative RT-PCR

To verify the results of RNA sequence, total RNA from the control, PGCC (at recovery day 3), and daughter cells were extracted from tested cell lines as reported previously [7]; Primers were designed according to reference [28] and listed in Table S1 [29].

### ELISA

Supernatants of Hey cell cultures without FBS were collected at different intervals (0–30 recovery days) following treatment with paclitaxel and evaluated using a human Duoset® ELISA kit for IL-1β, IL-6, IL-8, Gro-α, tumor necrosis factor (TNF)-α, granulocyte-macrophage colony-stimulating factor (GM-CSF; R&D Systems), respectively, according to the manufacturers’ protocols.

### Inhibition of proinflammation, propidium iodide staining, and flow cytometry

Inhibitors of inflammation–IL-6R antibody (0–5 μg/ml, R&D Systems) and apigenin (0-20 μM, Sigma) were incubated with Hey cells individually and in combination with and without paclitaxel (500 nM) overnight, and paclitaxel was removed, and cells were cultured in regular medium for another 24–48 h. On recovery day 3, supernatant and cells were collected respectively. DNA content (≥4 C are defined as PGCCs) was analyzed as previously described [6]. Secretion levels of IL-1β, IL-6, IL-8, Gro-α, TNF-α, and GM-CSF (also known as CSF2, colony-stimulating factor 2) in the test supernatant (within 24 h) were detected with the ELISA kit as described above.

### Immunofluorescence and confocal scanning

To study the expressions of proteins OCT4, NANOG, and SOX2 in PGCCs, and the expression and distribution of collagen I and alpha-smooth muscle actin (α-SMA) in fibroblasts, immunofluorescence and confocal scanning were performed as previously described [7]. Primary antibodies are listed in Table S2.

### Western blotting

Western blot analyses to detect the embryonic markers in cancer cells and expression levels of VEGF, IL-6R, p-STAT3, and STAT3 in fibroblasts were carried out at different time points as described previously [6]. Details of the primary antibodies are listed in Table S2.

### Primary culture of fibroblasts and coculture of PGCCs and fibroblasts

Fibroblasts were isolated from normal human fallopian tubes and cultured as described previously [12]. PGCCs (recovery day 7, marked as PGCCs Sup) or corresponding test cells (regular cancer cells, named as group of Reg-sup) were grown in the inserts (pore size 0.4 µm; BD Falcon, Bedford, MA, USA), together with the primary fibroblasts for 72 h in the medium of tested groups. Then the supernatant, total RNA, and protein of fibroblasts were collected, respectively for downstream experiments [29]. A subpopulation of GPR77 + /CD10 + fibroblasts was quantified with flow cytometry using direct antibodies as described previously [30].

### Blockage of IL-6 function in PDX models

56 SCID male mice (3-week-old) bearing MDACC-HGSC-1 or Hey ovarian cancer xenografts were treated intraperitoneally with PBS (500 µl, containing 10% Cremophor EL as control, Sigma), paclitaxel (6 µg/g in 500 µl), IL-6R antibody (tocilizumab, 50 µg/g in 500 µl), apigenin (25 µg/g in 500 µl), paclitaxel + IL-6R antibody, paclitaxel + apigenin, or paclitaxel + IL-6R antibody + apigenin twice a week for 8 weeks. Tumor sizes were recorded weekly. After 8 weeks of treatment, the mice were sacrificed, and the tumors were collected for H&E staining and immunohistochemistry (IHC) for Collagen I and CD31 as reported previously [23].

### Analysis of human high-grade serous carcinoma tissue

Thirty-eight paired (pre- and postneoadjuvant chemotherapy treatment with carboplatin and paclitaxel) HGSC samples were randomly selected from the MD Anderson ovarian tumor registry. The prechemotherapy samples were used as the control group. H&E staining and IHC for collagen I and CD31 were performed on all 38 cases to observe histologic features and analyze the proportion of PGCCs as described previously [6].

### Statistical analysis

All results of bar graphs were represented as the mean ± SD obtained from at least three independent experiments. Statistical differences were evaluated using the Student’s *t* test, one-way analysis of variance, or *χ*^2^ analysis as appropriate with SPSS 13.0 (SPSS Inc, Chicago, IL, USA). P ≤ 0.05 was considered statistically significant.

## Results

### Initiation of PGCCs is associated with activation of a cytokine storm

**Fig. 1:**
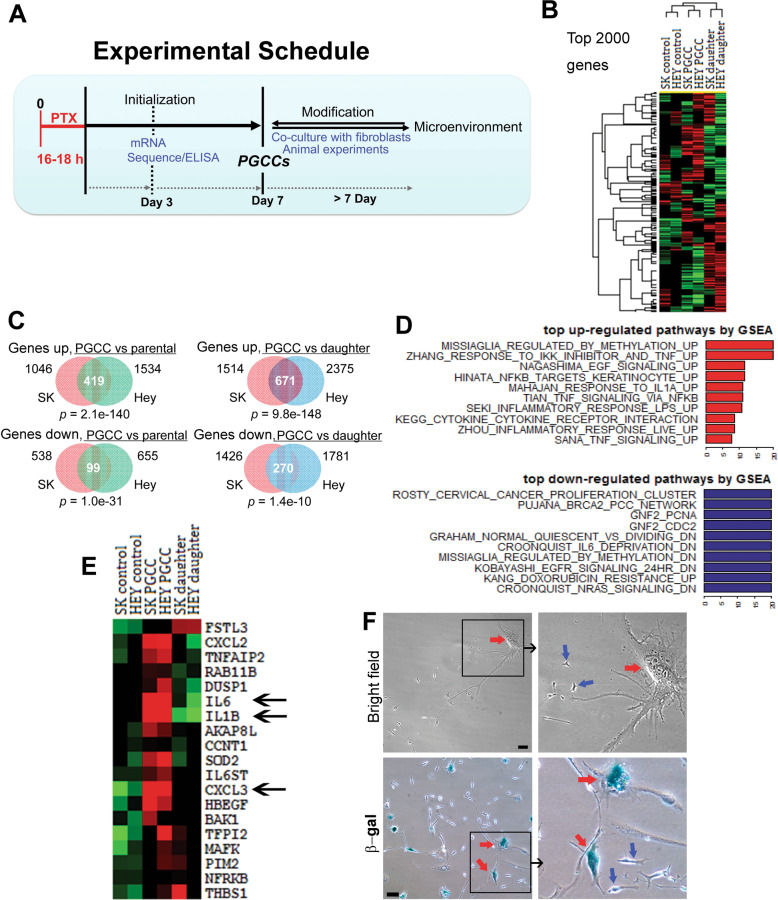
Paclitaxel treatment triggers inflammatory response. **A** Experimental design. After treatment with paclitaxel (PTX; 500 nM) overnight (16-18 h), ovarian cancer cells, including the control cells without treatment and PGCCs induced by PTX were allowed to recover in their corresponding regular media. From recovery day 1 to 30, proinflammatory molecule levels were measured in the supernatant every 24 h. Total mRNA for sequencing was collected at recovery day 3. PGCCs collected at recovery day 7 were used for the coculture system with primary fibroblasts. **B** Heat map of the top 2000 genes induced by paclitaxel treatment of Hey and SKOv3 cells (control, PGCCs at recovery day 3, and daughter cells). **C** Up- or downregulated genes in PGCCs of Hey and SKOv3 cells (at recovery day 3) compared with the control and daughter cells. **D** The top up- or downregulated pathways in PGCCs compared with the control cells. **E** Top inflammatory molecules that were upregulated in PGCCs, among which IL-6 and IL-1β, along with CXCL3, were the predominant molecules (arrows). **F** Representative pictures of daughter cells (blue arrows) from mother PGCCs (red arrows, at recovery day 21). PGCCs are positive for β-gal staining (lower panels, at recovery day 21), which indicates senescence. Bars, 50 µm.

To elucidate potential mechanism(s) of PGCC initiation, we performed mRNA expression analysis via RNA-seq. The experimental design is shown in Fig. 1A. Following treatment with paclitaxel, the total mRNA of PGCCs was collected for RNA-seq at recovering day 3 [7]. PGCCs at recovery day 7 were used for coculture with fibroblasts. The supernatant and protein of PGCCs were also collected for downstream ELISA and Western blot analysis.

As expected, treatment with paclitaxel led to mitotic crisis and massive cell death; it also led to the formation of PGCCs (Figure S1). The optimal concentration of paclitaxel for inducing PGCCs was 500 nM for Hey and SKOV3 cells, as we have previously reported [7], and 1000 nM for the PDX cell line MDACC-HGSC-1 (Figure S1A and B). Although many PGCCs were found to be senescent [6], 45.2 ± 8.5% (mean ± SD) of PGCCs were viable at recovery day 7 (Figure S1C. Q3, viable subgroup; Q2 + Q4, apoptotic subgroup). Small daughter cells budded out from the mother PGCCs and formed clones after recovering for 14–21 days (Figure S1D).

RNA-seq analysis showed that more than 2000 genes were up- or downregulated in PGCCs on recovery day 3 (Fig. 1B). When compared to the parental cells, there were 419 upregulated and 99 downregulated genes in PGCCs in both the Hey and SKOV3 cell lines (Fig. 1C). When compared with daughter cells, there were 671 upregulated and 270 downregulated genes in PGCCs in both the cell lines tested. When pathways were analyzed, the inflammatory response, including TNF-α, NF-κB, Lipopolysaccharide (LPS), and cytokines were the top upregulated pathways in PGCCs (Fig. 1D), while proliferation and cell-division-related pathways were inhibited (Fig. 1D). Among the proinflammatory molecules, IL-6 was the most predominant factor upregulated in PGCCs (Fig. 1E). Moreover, together with inflammation burst, PGCCs (at recovery day 7) were found to go through senescence (PGCCs, 85 ± 7.4%; regular cancer cells, 3.8 ± 2.1%; daughter cells, 9.4 ± 3.4%), as indicated by positive β-gal staining (Fig. 1F). These results demonstrated that the inflammatory response, dominated by IL-6 and the activation of senescence are associated with the development of PGCCs.

**Fig. 2:**
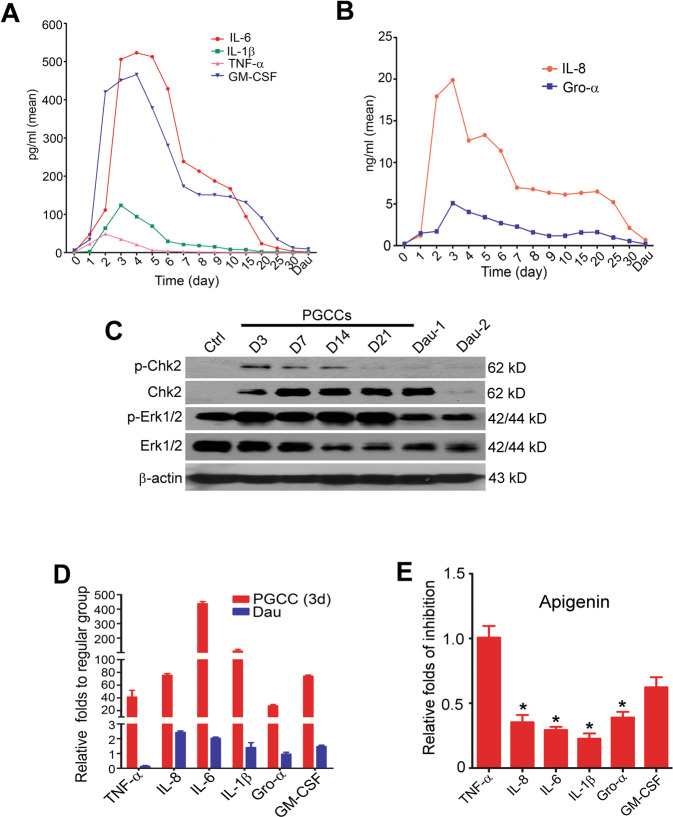
ELISA and Western blot verification of inflammation burst triggered by paclitaxel. **A**, **B** ELISA verification of the increased production (within 24 h) of inflammatory molecules including IL-6 (peak at recovery day 4, 522.9 pg/ml), IL-1β (peak at recovery day 3, 123.5 pg/ml), TNF-α (peak at recovery day 2, 48.9 pg/ml), GM-CSF (peak at recovery day 4, 465.6 pg/ml), IL-8 (peak at recovery day 3, 19.8 ng/ml), and Gro-α (peak peak at recovery day 3, 5.1 ng/ml) at different recovering time point. **C** Phosphorylation of Chk2 and Erk1/2 was activated by paclitaxel exposure and kept at a high level for the first two recovering weeks, which was consistent with the elevated inflammatory molecules shown in (**A**) and (**B**). **D** Fold increases (via ELISA) in TNF-α (41.2 ± 10.6), IL-8 (75.4 ± 2.7), IL-6 (437.9 ± 15.1), IL-1β (110.6 ± 11.6), Gro-α (27.4 ± 2.2), and GM-CSF (73.9 ± 1.8) by PGCCs (at recovery day 3) compared with the control (regular group). **E** The inflammatory burst at recovery day 3 was inhibited by apigenin. Levels of TNF-α, IL-8, IL-6, IL-1β, Gro-α, and GM-CSF decreased by 1 ± 0.1, 0.35 ± 0.06, 0.29 ± 0.03, 0.23 ± 0.04, 0.39 ± 0.04, and 0.6 ± 0.08 folds, respectively, compared with the control. *P *<* 0.05.

We further validated the mRNA sequencing data with RT-qPCR (Figure S2), ELISA, and Western blotting (Fig. 2) in tested cell lines. The expression of representative protein was found to be consistent with the RNA-seq data (Figure S2). For the inflammatory burst, we tested the levels of TNF-α, IL-8, IL-6, IL-1β, Gro-α, and GM-CSF produced by PGCCs within 24 h throughout the 30-day recovery period (Fig. 2A and B). Following paclitaxel administration, production of these inflammatory molecules began to increase immediately. Their levels reached a peak at recovery day 2–4 and sustained a high level for the following 1–2 weeks of the PGCC life span. Phosphorylation of Chk2 and Erk 1/2 proteins was activated as the inflammation response waxed and waned (Fig. 2C). Two close bands of Chk2 were found, which was commonly seen in activation of inflammatory cascade, due to the isoform or glycosylation [31]. On recovery day 3, levels of TNF-α, IL-8, IL-6, IL-1β, Gro-α, and GM-CSF were increased by 41.2 ± 10.6-, 75.4 ± 2.7-, 437.9 ± 15.1-, 110.6 ± 11.6-, 27.4 ± 2.2-, and 73.9 ± 1.8-fold, respectively, compared with the control (Fig. 2D). Notably, IL-6 was the most predominantly increased inflammatory molecule that has been found to be involved in tumorigenesis and cancer stemness and predominantly expressed in PGCCs rather than in diploid cancer cells [32–34]. Apigenin is a plant bioactive compound that was found to be effective to prevent a wide range of chronic diseases including cancer, diabetes, and stroke. In this study, we employed apigenin as a parallel medicine with IL-6 antibody to investigate the role of inflammation in early events of PGCCs development. The production of IL-6, IL-8, IL-1β, and Gro-α was inhibited significantly by apigenin (Fig. 2E). These data indicate that an inflammation burst dominated by IL-6 was involved in the development of PGCCs.

### IL-6 is involved in activation of embryonic stemness program in PGCCs

**Fig. 3:**
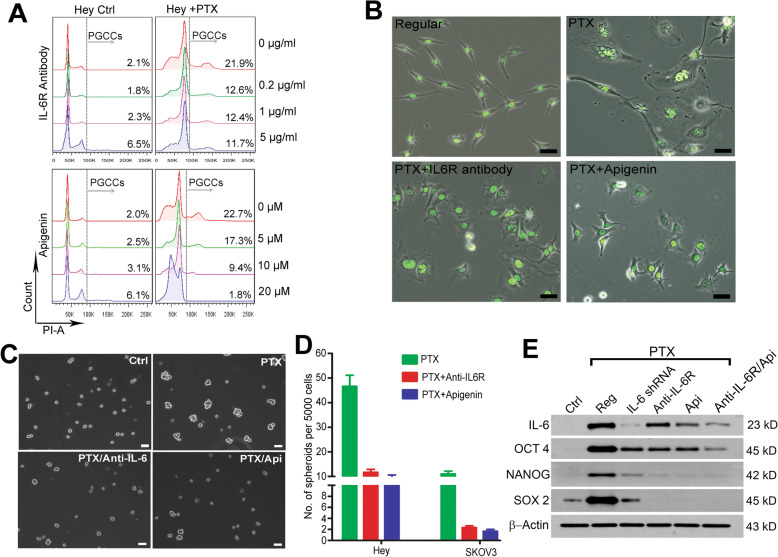
Inflammation response facilitates the development and stemness of PGCCs. **A**, **B** PGCC formation (at recovery day 3) was inhibited by IL-6R antibody and apigenin in a dose-dependent manner, which was confirmed by population percentage (**A**, via flow cytometry; PGCCs, DNA content>4 **C** as grouped by grey arrows.) and morphological observation (**B**, DNA was labeled with H2B-GFP). Bars, 50 µm. **C, D** Spheroid formation (>=200 µm in diameter) capability of PGCCs was inhibited by apigenin (Api) and IL-6R antibody at day 7. In Hey cells, the number of spheroids developed after treatment with paclitaxel (PTX), PTX + anti-IL-6R, and PTX + Api were 46.7 ± 7.7, 11.7 ± 2.1, and 10.5 ± 1.1, respectively. In SKOV3 cells, the number of spheroids in each subgroup was 11.0 ± 2.0, 2.3 ± 0.5, and 1.7 ± 0.6, respectively. Bars, 200 µm. **E** Expression of embryonic markers including OCT4, NANOG, and SOX2 in PGCCs (recovery day 7) was downregulated by IL-6 knockdown (IL-6 shRNA), blocking with IL-6R antibody, apigenin, and IL-6R antibody + Api. Bars, 50 µm.

To determine the pleiotropic roles of IL-6 in stemness activation of PGCCs, we blocked the IL-6 feedback loop in PGCCs with IL-6R antibody and/or apigenin. Compared with the control (20.5 ± 4.6%), blocking the IL-6 pathway with IL-6R antibody reduced the PGCC percentage to 12.1 ± 3.3%, 11.8 ± 3.8%, and 10.5 ± 2.1% at the concentrations of 0.2, 1, and 5 µg/ml, respectively (Fig. 3A. PGCCs, DNA content *n* ≥ 4 C). Blocking the cytokine storm with apigenin also led to significant decreases in the PGCC percentage, to 17.1 ± 2.2, 9.5 ± 1.9, and 1.5 ± 0.5% at concentrations of 5, 10, and 20 µM, respectively. The inhibition of PGCC formation was further confirmed by morphology (Fig. 3B). These studies demonstrated that an early inflammatory response, induced by paclitaxel and dominated by IL-6, promoted PGCC development, which was inhibited by IL-6R antibody and/or doses of IL-6R antibody at 5 µg/ml and apigenin at 10 µM were selected for the following subsequent experiments.

We previously found that PGCCs could form spheroids in stem cell medium and acquired embryonic stemness marked by expression of OCT4, NANOG, and SOX2, which are involved in ovarian cancer relapse. Compared with the control, more compacted spheroids (diameter ≥ 200 µm) were developed from PGCCs, with bigger size and embryonic features such as cleavage [6]. We, therefore, next examined the effect of the inflammatory burst on the activation of stemness in PGCCs by measuring their ability to form spheroids and the expression levels of OCT4, NANOG, and SOX2. Compared with the control group (8 ± 3 per 5000 cells in 3 ml medium), there were 46.7 ± 7.7, 11.7 ± 2.1, and 10.0 ± 1.5 spheroids in the paclitaxel, paclitaxel + IL-6R antibody, and paclitaxel + apigenin groups, respectively (Fig. 3C and D). Blockage of IL-6 activity by IL-6R antibody or apigenin significantly reduced spheroid formation by 75% and 79.6%, respectively. This indicates inflammation predominated by IL-6 promote the formation of spheroids from PGCCs. Production of IL-6 in PGCCs was downregulated by IL-6 shRNA, IL-6R antibody, apigenin, or IL-6R antibody and apigenin combined at different levels (Fig. 3E), which suggests that IL-6 function in PGCCs is controlled in an autocrine feedback manner. Levels of NANOG and SOX2 were much more affected by IL-6 inhibition than that of OCT4. Blocking the IL-6 loop with IL-6R antibody and apigenin extracellularly was more efficient than knocking down the production of IL-6 intracellularly with shRNA.

### IL-6 increases collagen production, the GPR77 + /CD10 + population of fibroblasts, and VEGF expression in fibroblasts

We used a coculture system of PGCCs (supernatant of PGCCs, PGCCs Sup), the corresponding regular cancer cells (supernatant of regular cancer cells, Reg Sup), and fibroblasts in vitro that mimics the interaction of PGCCs and fibroblasts in vivo. Morphologic changes in the fibroblasts were apparent in the test groups of PGCCs Sup and IL-6 (Figure S3A). In the presence of PGCCs alone or IL-6 protein alone, fibroblasts showed slow growth with increased cell size and abundant cytoplasm, similar to that observed with paclitaxel treatment (Figure S3A). However, the development of polyploidy in fibroblasts was induced by paclitaxel treatment alone (13.7 ± 1.5 polyploid cells per 5000 cells compared to 5.8 ± 0.4 polyploid cells in the control group) and not by PGCCs Sup or IL-6 (Figure S3B).

**Fig. 4:**
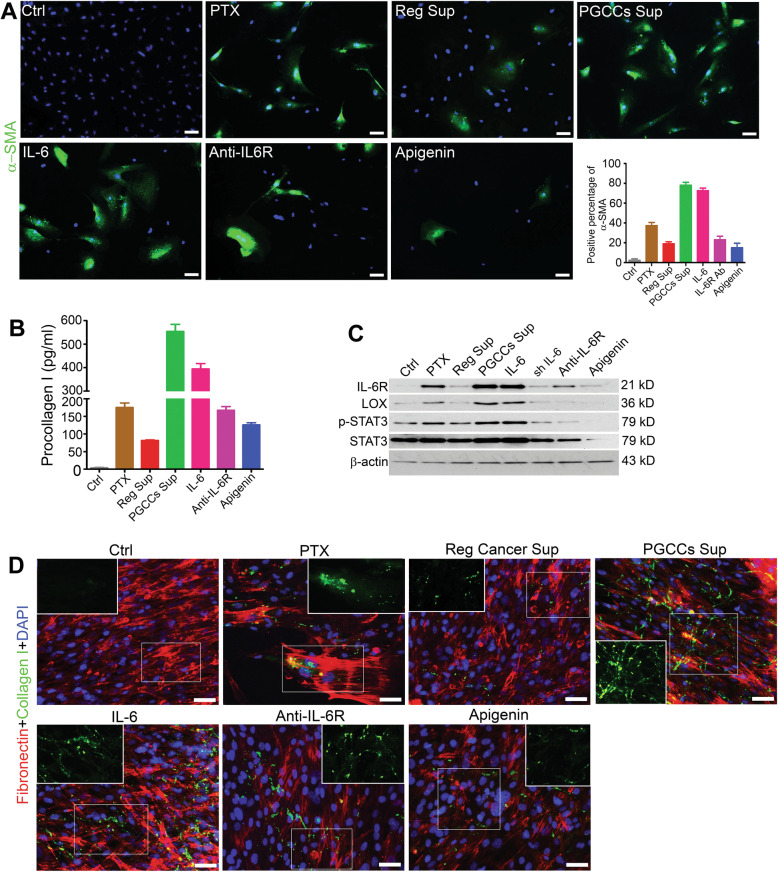
PGCCs facilitate transformation of CAFs and stimulate production of collagen I by fibroblasts dominantly via IL-6 pathway. **A** Coculture of PGCCs or IL-6 with normal fibroblasts (for 72 h) enriched the population of CAFs (indicated as α-SMA positivity), which can be inhibited by IL-6R antibody and apigenin. Positive percentage of α-SMA in test groups of fibroblasts: Control, 2.3 ± 1.5%; paclitaxel (PTX), 37.3 ± 3.2%; Reg Sup, 19.0 ± 2.1%; PGCCs Sup, 78.0 ± 3.0%; IL-6, 72.7 ± 2.5%; IL-6R antibody, 23.0 ± 3.6%; apigenin, 15.0 ± 4.6%. Bars, 50 µm. **B** Level of procollagen I in fibroblasts (cocultured for 72 h) was significantly triggered by PGCCs Sup (554.3 ± 29.7 pg/ml) and IL-6 protein (394.3 ± 23.1 pg/ml) and reduced by blocking with IL-6R antibody (167.3 ± 10.7 pg/ml) and apigenin (126.5 ± 6.4 pg/ml). **C** LOX level and phosphorylation of STAT3 in fibroblasts (cocultured for 72 h) was increased when exposed to PGCCs, IL-6, and PTX and was inhibited by IL-6 knockdown, IL-6R antibody, and apigenin. **D** Cross-linking and deposition of collagen I outside fibroblasts (cocultured for 72 h) were significantly apparent and web-like when cocultured with PGCCs or treated by IL-6 protein alone. This cross-linking was attenuated by IL-6R blockage and apigenin. When treated with PTX only, the collagen is cluster- or spot-like. Panel corners show higher magnification of the squared areas to clearly show the morphology of collagen. Bars, 50 µm.

To examine the phenotypic conversion of fibroblasts in response to secretions from PGCCs or IL-6 alone, we performed immunofluorescence staining for α-SMA, a commonly used marker for cancer-associated fibroblasts (CAFs). As shown in Fig. 4A, the expression of α-SMA was significantly increased in PGCCs Sup and IL-6 (Control, 2.3 ± 1.5%; Paclitaxel, 37.3 ± 3.2%; Reg Sup, 19.0 ± 2.1%; PGCCs Sup, 78.0 ± 3.0%; IL-6, 72.7 ± 2.5%). Neutralization of IL-6 with IL-6R antibody (23.0 ± 3.6%) and apigenin (15.0 ± 4.6%) decreased the α-SMA positivity in fibroblasts. The above data suggest that PGCC-derived IL-6 was the critical factor in transforming fibroblasts into CAFs. In addition, as shown in Fig. 4B, we found that PGCCs Sup (554.3 ± 29.7 pg/ml) or IL-6 protein (394.3 ± 23.1 pg/ml) stimulated synthesis of procollagen I (dissoluble precursor of collagen I) in the fibroblasts, which was inhibited by IL-6R antibody (167.3 ± 10.7 pg/ml) and apigenin (126.0 ± 6.1 pg/ml) (Fig. 4B). We also examined the expression of procollagen I’s rate-limiting enzyme for collagen synthesis, lysyl oxidase (LOX), and we found that the expression of LOX was markedly increased by paclitaxel, PGCCs Sup, and IL-6 protein and that these increases could be blocked by shRNA, IL-6R antibody, and apigenin (Fig. 4C). STAT3, a critical downstream protein for the IL-6/IL-6R pathway, was found to be highly phosphorylated in the test groups mentioned above (Fig. 4C). These data indicate that IL-6/IL-6R signaling is activated in PGCCs, which in turn can activate STAT3 phosphorylation and facilitate transformation into CAFs via the synthesis of collagen by transformed fibroblasts and activation of LOX.

The cross-linking and remodeling of collagen after secretion plays a critical role in collagen maturation and function in the TME. Thus, we further examined collagen cross-linking and deposition around the fibroblasts (Fig. 4D and Figure S3C). Compared with controls, fibroblasts treated with paclitaxel had more cluster and spot-like collagen I outside the fibroblasts, indicating that paclitaxel exposure can trigger the production and deposition of collagen I from fibroblasts. Compared with the control and Reg Sup groups, fibroblasts incubated with PGCCs Sup or IL-6 protein only developed more web-like collagen I with a greater degree of cross-linking, which was attenuated by IL-6R antibody and apigenin. These results show that the IL-6/IL-6R pathway initiated by PGCCs (induced by paclitaxel), rather than paclitaxel per se or regular cancer cells, plays a key role in the organization and remodeling of collagen I, and this remodeling can be attenuated by blocking IL-6.

**Fig. 5:**
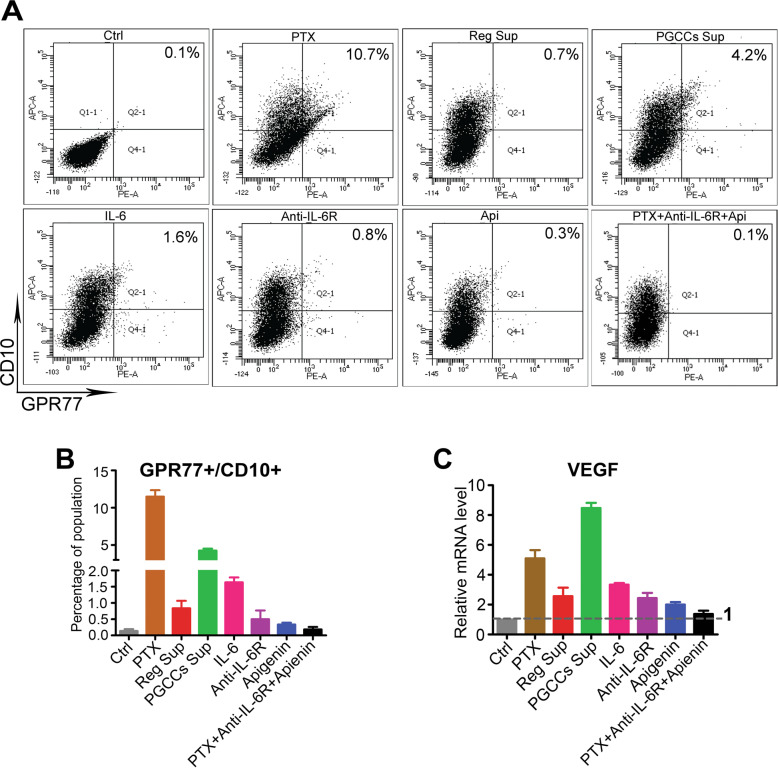
PGCCs and IL-6 enriched the GPR77 + /CD10 + population and increased VEGF expression of fibroblasts. **A**, **B** GPR77 + /CD10 + population in fibroblasts increased significantly after treatment with paclitaxel (PTX; 11.5 ± 0.9%), PGCCs Sup (4.3 ± 0.3%), and IL-6 (1.6 ± 0.2%) compared with the control (0.1 ± 0.1%). **C** Expression of VEGF in fibroblasts was elevated significantly in the subgroups of PGCCs Sup (8.5 ± 0.4-fold), PTX (5.1 ± 0.6-fold), and IL-6 (3.3 ± 0.1-fold), respectively, which was inhibited by IL-6R antibody (2.4 ± 0.4-fold), apigenin (2.0 ± 0.2-fold), or combination (1.4 ± 0.2-fold).

The GPR77 + /CD10 + subpopulation of fibroblasts is critical for maintaining the stemness of cancer cells by forming a niche microenvironment around the cancer cells. To determine whether PGCCs affect the expression of GPR77 and CD10, we examined the expression of these two markers in the fibroblasts in the presence or absence of treatment. As shown in Fig. 5, the GPR77 + /CD10 + population of fibroblasts in the PGCCs Sup alone (4.3 ± 0.3%) and IL-6 protein alone (1.6 ± 0.2%) groups increased significantly, by 43-fold and 16-fold, respectively, compared with the control (0.1 ± 0.1%, Fig. 5A and B). This GPR77 + /CD10 + phenotype was blocked in the presence of IL-6R antibody, apigenin, and their combination (Fig. 5A and B). The expression of VEGF, a well-described angiogenic factor, was also elevated by 5.1-, 8.5-, and 3.3-fold in the paclitaxel, PGCCs Sup, and IL-6 protein groups, respectively (Fig. 5C), and by 2.6-, 2.4-, and 2.0-fold in the Reg Sup, IL-6R antibody, and apigenin groups. These results suggest that PGCC-derived IL-6 may help to maintain the stemness of PGCCs by enriching the GPR77 + /CD10 + population of fibroblasts and activating angiogenesis via VEGF.

### Anti-IL-6R antibody and apigenin reduce PGCC formation and decrease tumor growth in PDX models

**Fig. 6:**
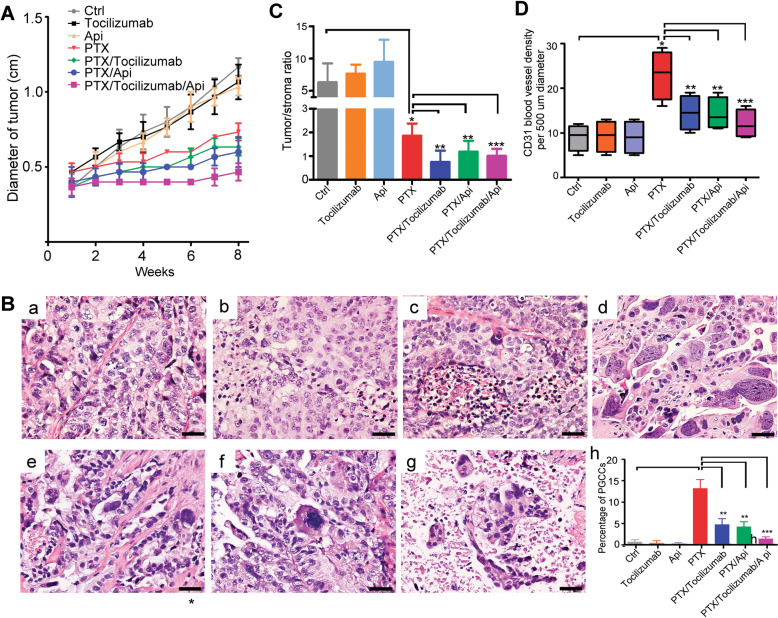
Inflammation and IL-6 triggered by paclitaxel exposure facilitate tumor growth and modification of the tumor microenvironment in vivo. **A** Tumor-proliferation curve. Inflammation attenuation and IL-6R blocking (with tocilizumab) enhanced the tumor-proliferation inhibition of paclitaxel (PTX). **B** Histology features (a-g) and PGCC percentage (h) of tumor in each group. Compared with the control (1.8 ± 0.7%), PGCCs were significantly induced by PTX alone (14.6 ± 5.4%, *P* < 0.05), which was attenuated by combined administration of PTX with tocilizumab (5.2 ± 2.1%, *P* < 0.05) and/or apigenin (4.8 ± 1.9%, *P* < 0.05), but tocilizumab or apigenin alone had no significant effect. H&E staining, bars, 50 µm. a, control; b, tocilizumab; c, apigenin; d, PTX; e, PTX + tocilizumab; f, PTX + apigenin; g, PTX + tocilizumab + apigenin. **C** PTX can significantly lower the tumor/stroma ratio (Control, 6.3 ± 2.9; PTX, 1.9 ± 0.5), which can be enhanced by tocilizumab (0.8 ± 0.5) and apigenin (1.2 ± 0.5). **D** Microvessel density in tumor increased significantly in the subgroups of PTX (23 ± 5.5 per area of 500 µm diameter), PTX + tocilizumab (14.5 ± 3.9), PTX + apigenin (14.3 ± 3.6), and PTX + tocilizumab + apigenin (12.1 ± 3.2). IL-6R blocking and apigenin reduced the effect of PTX alone when combined together with PTX.

To investigate the effect of IL-6 blocking on tumor growth in vivo, we administered tocilizumab (IL-6R antibody) and apigenin, individually or together with paclitaxel, to mice bearing PDX tumors. Compared with the control group, tocilizumab or apigenin alone did not significantly affect tumor growth (Fig. 6A), but paclitaxel alone and combined with tocilizumab, apigenin, or both the inhibited tumor growth significantly (Fig. 6A). Compared with paclitaxel alone, paclitaxel + tocilizumab + apigenin inhibited tumor growth most effectively.

Histologically, paclitaxel treatment led to the formation of PGCCs with bizarre nuclei (Figure 6Ba-g) and abundant collagen deposits in the stroma (Figure S4A). Compared with the control (1.8 ± 0.7%), PGCCs were significantly induced by paclitaxel (14.6 ± 5.4%), which was inhibited by co-administration of tocilizumab (5.2 ± 2.1%), apigenin (4.8 ± 1.9%), or both (2.5 ± 0.8%) (Figure 6Bh). The tumor/stroma ratios in the paclitaxel, paclitaxel + tocilizumab, paclitaxel + apigenin, and paclitaxel + tocilizumab + apigenin groups were 1.9 ± 0.5, 0.8 ± 0.5, 1.2 ± 0.5, and 1.0 ± 0.3, respectively, which were significantly lower than the ratios in the control (6.3 ± 2.9), tocilizumab only (7.7 ± 1.4), and apigenin only (9.5 ± 3.4) groups (Fig. 6C and D, Figure S4B). MVD in tumor stroma was 23 ± 5.5, 14.5 ± 3.9, 14.3 ± 3.6, and 12.1 ± 3.2 microvessels /500 µm diameter in paclitaxel, paclitaxel + tocilizumab, paclitaxel + apigenin, and paclitaxel + tocilizumab + apigenin, respectively, which was significantly higher than in the other three groups (control, tocilizumab only, apigenin only. Figure S4C, Fig. 6D). Tocilizumab and apigenin, alone or in combination, combined with paclitaxel can significantly decrease the percentage of PGCCs, tumor/stroma ratio, and MVD in tumor compared with paclitaxel alone. These results suggest that blockage of PGCC-derived IL-6 can enhance the therapeutic effect of paclitaxel by modifying the TME in terms of PGCC formation, collagen production, and angiogenesis.

### Paclitaxel treatment increases collagen deposition, microvascular density, and the GPR77+/CD10+population of fibroblasts in human ovarian cancers

**Fig. 7:**
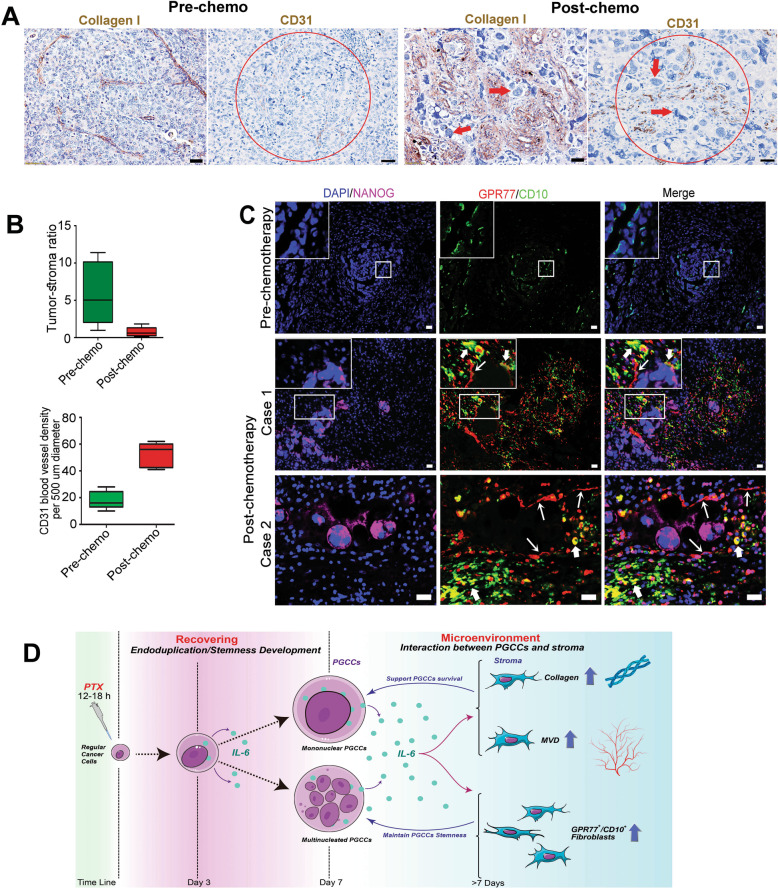
Microenvironment events in human ovarian cancer before and after paclitaxel treatment. **A** Representative pictures of stroma (collagen I) and microvessel density (CD31) in paired tumors before and after chemotherapy. PGCCs are indicated by the red arrows. **B** The tumor/stroma ratio decreased (prechemo, 5.9 ± 1.9; postchemo, 0.7 ± 0.3; upper panel) while microvessel density increased (prechemo, 18.2 ± 6.7; postchemo, 52.2 ± 9.2; lower panel) in the tumor after chemotherapy. **C** NANOG (representative embryonic stemness marker) and the population of GPR77 + /CD10 + fibroblasts in human tumor stroma were increased after paclitaxel treatment. Bars, 50 µm. **D** Schematic of the role of IL-6 in PGCC formation and the interaction between PGCCs and fibroblasts. Stressed by paclitaxel (PTX) treatment, cancer cells go through endoduplication to form PGCCs (mononuclear or multinucleated), and an inflammation response, predominantly IL-6, was triggered. Autocrine IL-6 plays a critical role in the development of PGCCs. IL-6 produced by PGCCs promotes the transformation of fibroblasts to synthesize collagen and microvessels, which can support the survival of PGCCs. IL-6 derived from PGCCs can also enrich the GPR77 + /CD10 + population of fibroblasts, which can maintain the stemness of PGCCs.

To determine whether the TME is similarly changed following chemotherapy in human ovarian cancer, we examined the percentage of PGCCs and stromal changes (marked with collagen I) in 38 paired pre- and postchemotherapy cases. The percentage of PGCCs increased from 23.7% (9/38) before chemotherapy to 65.7% (25/38) after chemotherapy (*P* < 0.05; Fig. 7A, red arrowheads). The tumor/stroma (collagen I) ratio dramatically decreased from 5.9 ± 1.9 prechemotherapy to 0.7 ± 0.3 postchemotherapy (Fig. 7A and B), while MVD increased from 18.2 ± 6.7 prechemotherapy to 52.2 ± 9.2 postchemotherapy (Fig. 7A and B). The GPR77 + /CD10 + population of fibroblasts was also found to be mainly distributed around NANOG-positive PGCCs (Fig. 7C). These data suggest that paclitaxel treatment could promote PGCC formation and trigger IL-6 production, which is consistent with what we found in vitro and in vivo above.

## Discussion

In this study, we have identified the mechanism for the initiation of PGCCs and their role in generating therapeutic resistance: IL-6, a well-studied molecule in cancer biology and immunology, plays a critical role in the initiation of PGCCs and their communication with stromal reprogramming factors to acquire chemoresistance.

Paclitaxel and other chemotherapy drugs can damage the mitotic spindle and shut down mitosis, which lead a switch of mitotic cell cycle to endoreplication cell cycle and formation of PGCCs. These PGCCs acquire features of stemness and a proinflammatory secretory phenotype that contribute to the acquisition of chemoresistance [15]. The IL-6 pathway is essential for the transformation of fibroblasts into CAFs, which serve as fertile soil for tumor progression [26]. It has been shown that IL-6 alone is sufficient to convert non-stem cancer cells to cancer stem cells and thus expand the cancer stem cell population in breast and prostate cancerss via an IL-6 feedback loop [32, 35]. IL-6 has been shown to activate the expression of stem cell markers and confers chemotherapy resistance and predict poor prognosis in ovarian cancer [24, 25, 36]. Here, we found that IL-6 facilitates PGCC formation and the acquisition of embryonic stemness via an IL-6 autocrine loop, suggesting that embryonic stem cells are regulated through this autocrine loop. PGCCs can use IL-6 protein as a paracrine mechanism to facilitate the transformation of fibroblast to more tumor promoting CAFs for chemoresistance, supporting the established role that the TME plays a critical role in tumor development and chemoresistance.

PGCCs have recently been implicated in tumor initiation, resistance, and metastasis [4, 37–39]. The formation of PGCCs has been observed in multiple tumors, including ovarian cancer, breast cancer, and prostate cancer [27, 39, 40]. Several mechanisms have recently been described as mediating the therapeutic resistance of PGCCs, including their lipid-dependent metabolism [41] or promotion of tumor regrowth [39]. The anti-inflammatory inhibitors aspirin and resveratrol have been used in the prevention and clinical treatment of colorectal cancer via the elimination of tetraploid cells [42, 43], which can be considered as polyploid cells in cancer initiation. In addition, IL33 was found to promote the regular diploid tumor cells into PGCCs by snail deregulation and p53 inactivation [44, 45]. The formation, migratory and invasive features of PGCCs have been shown to be are regulated by inhibition of acid ceramidase, cytoskeletal organization and vimentin network [46, 47].

Our studies support our previous description in which the acquisition of resistance is mediated through the giant cell life cycle [7]. This process is initiated via activation of an inflammatory cytokine storm that leads to the activation of a stemness program for survival. The pleiotropic roles of IL-6 make it the most important mediator in the TME, forming a niche to favor the survival of PGCCs in two ways: (1) by acting on the PGCCs themselves, helping them to acquire polyploidy and embryonic stemness; and (2) by acting on stromal fibroblasts to modify the TME in terms of collagen production, VEGF expression of fibroblasts, and enrichment of the GPR77 + /CD10 + population. These effects both favor the survival and maintain the stemness of PGCCs.

On the basis of the above data and others [39, 44, 48, 49], we propose a model for paclitaxel-mediated therapeutic effect and mechanism of resistance as illustrated by Fig. 7D. Following the acute insult of paclitaxel, which leads to mitotic crisis and massive cancer cell death, the remaining cancer cells undergo genomic shock and activate a genomically imprinted emergency mechanism for survival [50–52]. This shock also leads to the activation of inflammation dominated by IL-6 and associated inflammatory network to reprogram both cancer cells and the stroma cells and creates microenvironment to promote the growth of newly reprogrammed daughter cells for the resistance and disease relapse.

Our data may have significant clinical implication. Anti-IL-6 or anti-IL-6R antibodies have been used as single agents in clinical trials in platinum-resistant patients [24]; however, these trials resulted in only modest effects in small numbers of patients. Our data have shown that once the drug-resistant cells become the dominant tumor mass, the IL-6 level largely returns toward low level, although in drug-resistant tumors, the IL-6 level may be higher than before chemotherapy. In addition, a single agent is unlikely to be effective, as the level of IL-6 is highest immediately after the administration of paclitaxel. Thus, anti-IL-6-based therapy is likely to be most effect when used together with paclitaxel or a combination of paclitaxel and carboplatin at the beginning of chemotherapy rather than for patients who have already acquired resistance. In addition, it will be helpful to select patients with a high level of IL-6R expression, which is likely to generate a cytokine storm following chemotherapy.

In summary, IL-6 may represent a key initiation event for the formation of PGCCs and creation of favorable tumor microenvironment in response to paclitaxel-mediated chemotherapy. This new biologic mechanism calls for clinical trials based on the beginning stage of giant cell life cycle and attack this novel adaptive mechanism for resistance, which may have the potential to improve outcomes of patients with ovarian cancers.

## Supplementary information

Supplementary Figures

Supplementary Tables
