# Supplementary material for: IL-6 promotes drug resistance through formation of polyploid giant cancer cells and stromal fibroblast reprogramming
Source: Oncogenesis. 2021 Sep 29;10(9):65. doi: 10.1038/s41389-021-00349-4 (PMC8481288; doi:10.1038/s41389-021-00349-4)
Supplement: Supplementary file 1 — Supplementary Figures [file 41389_2021_349_MOESM1_ESM.docx]

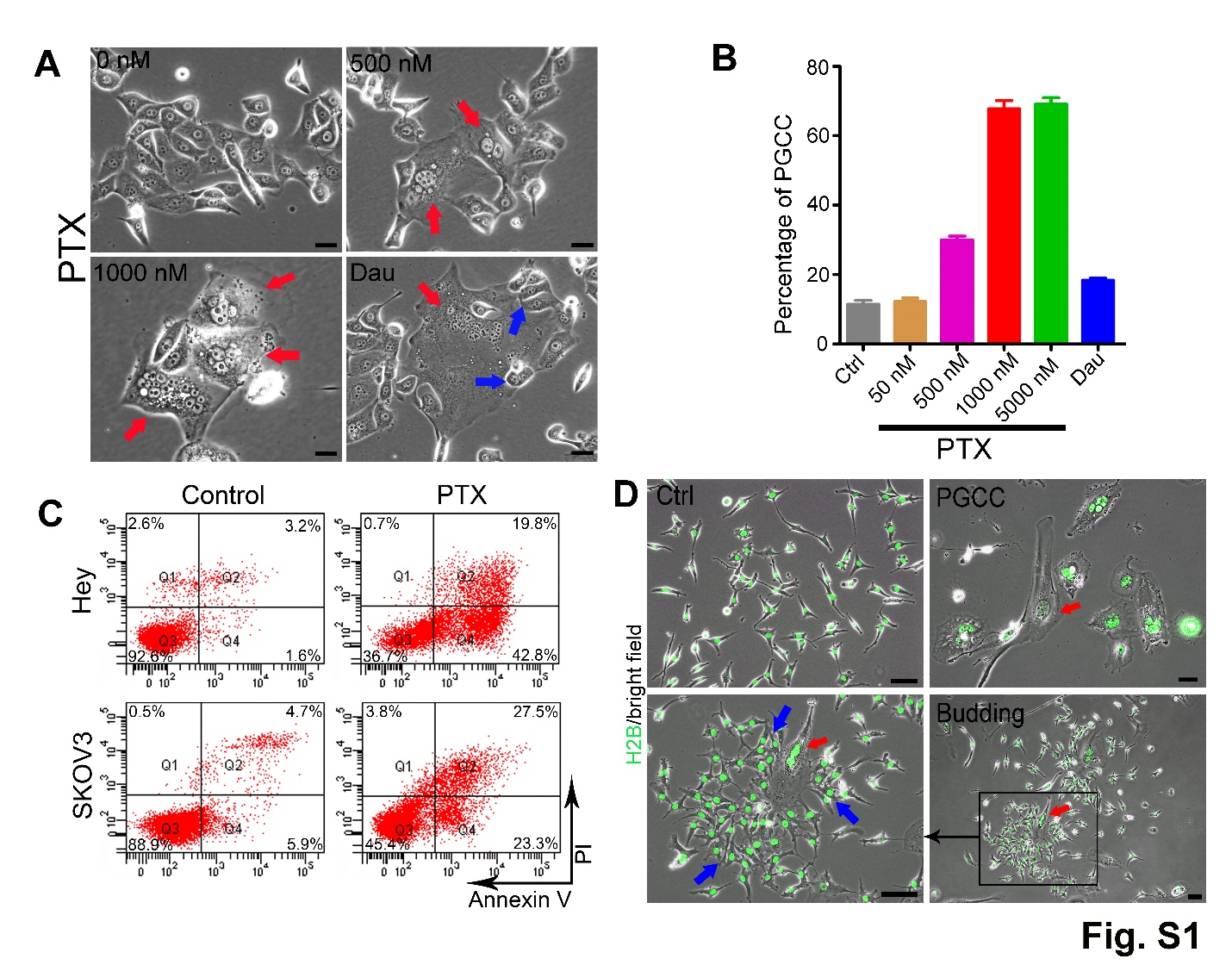


**Supplementary Figure 1. Morphology and viability of PGCCs at recovery day 7 after paclitaxel treatment.**

(A-B) The population of PGCCs gradually increased in MDACC-HGSC-1 cells in a dose-dependent manner. (A) PGCCs (red arrows) grew in clusters and upward. Small daughter cells (Dau, indicated with blue arrows) budded out and reverted to mitosis after recovering for two weeks (A, lower right panel). (B) After exposure to paclitaxel (PTX; 1000 nM), 75±3.7% of cancer cells became PGCCs (DNA content ≥ 4C, at recovery day 7).

(C) Viability analysis of PGCCs at recovery day 7 by Annexin V-PI assay via flow cytometry. Q3, viable subpopulation; Q2+Q4, apoptotic subpopulation.

(D) Representative photos of PGCCs labeled with H2B-GFP (the same single cell is indicated with red arrows) and PGCC-derived daughter cells (blue arrows) forming one single clone. The left lower panel is the magnification of the squared area.

Bars, 50 µm.


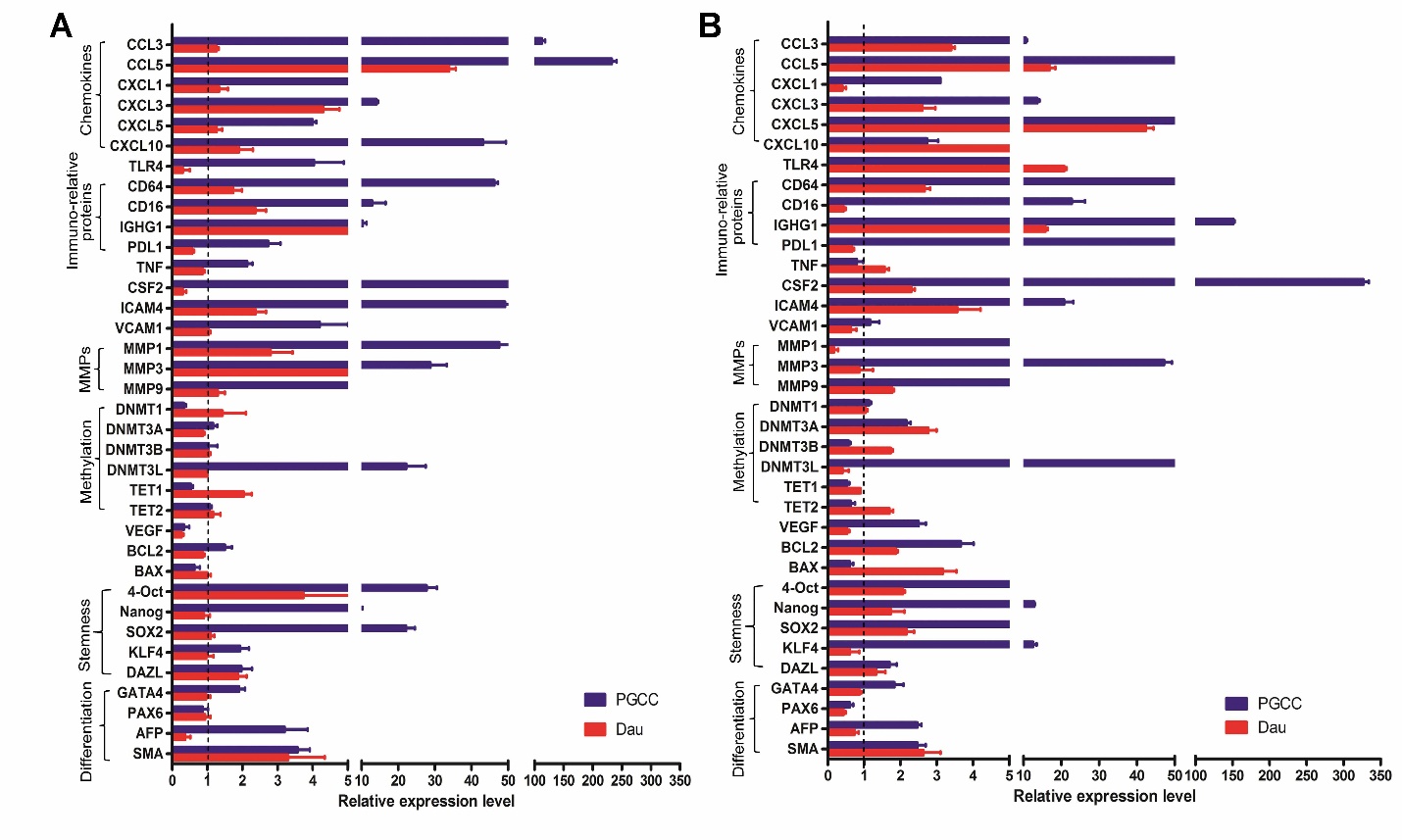


**Supplementary Figure 2. RT-PCR verification of mRNA expression**.

Expression levels of representative proteins in Hey (A) and SKOv3 (B) cells involved in inflammation, matrix metalloproteinase, methylation, stemness, and differentiation were verified in clusters by qRT-PCR.


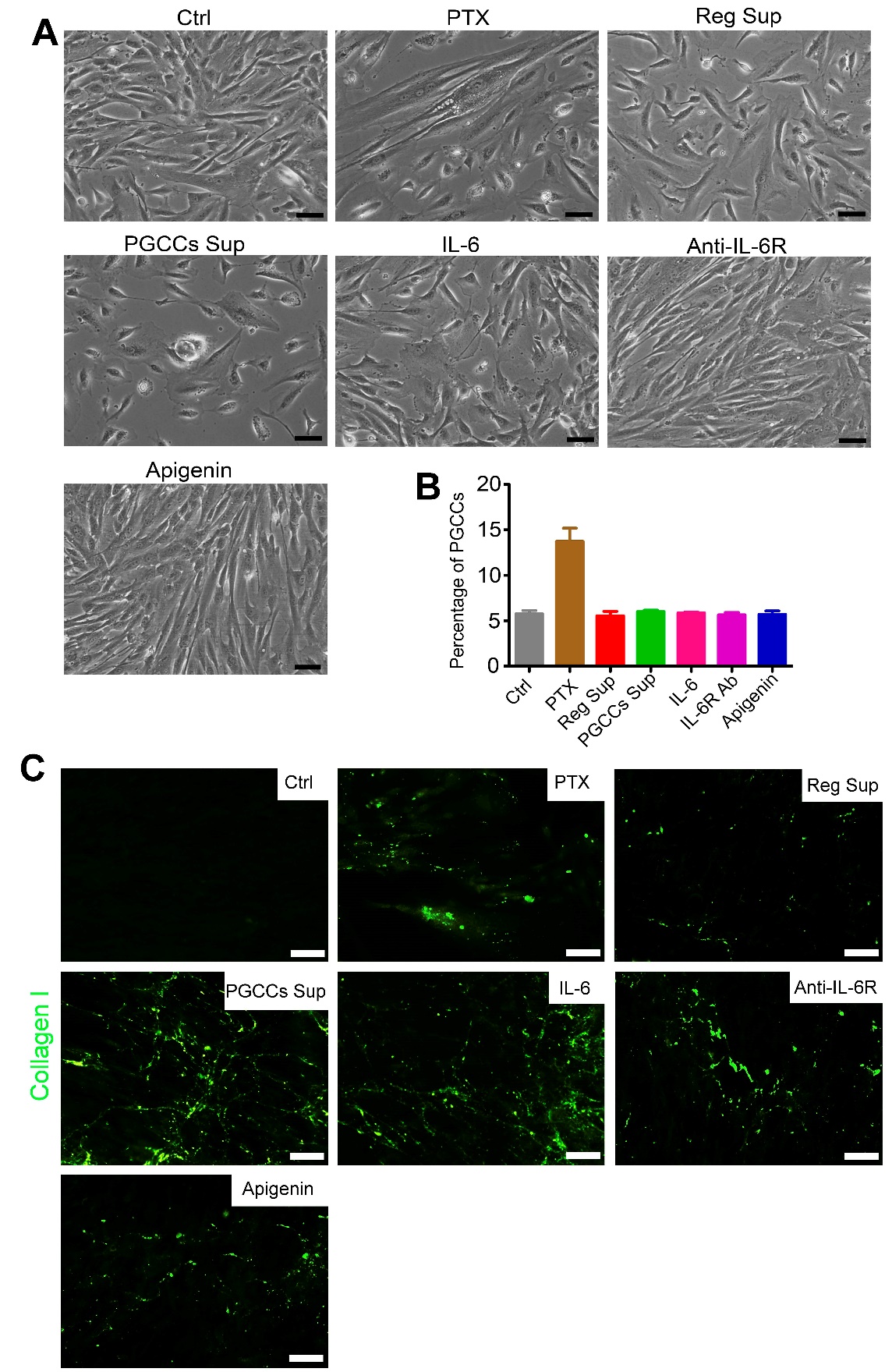


**Supplementary Figure 3. PGCCs and IL-6 facilitate transformation of fibroblasts and production and deposit of collagen I.**

(A, B) Fibroblasts enlarged in size and were rich in cytoplasm when co-cultured with PGCCs (PGCCs Sup) and treated with IL-6 protein alone. But compared with control (5.8±0.5%), there was no significant increase in the polyploidy percentage in these subgroups (PGCCs Sup, 6.2±0.2%; IL-6, 5.8±0.1%), except in the paclitaxel (PTX) group (13.7±1.5%) (B). Bars, 50 µm.

(C) Cross-linking and deposition of collagen I in each subgroup was more clearly apparent when only positive signals of collagen I were shown. PTX, cluster- or spot-like. PGCCs Sup and IL-6, web-like. Bars, 50 µm.


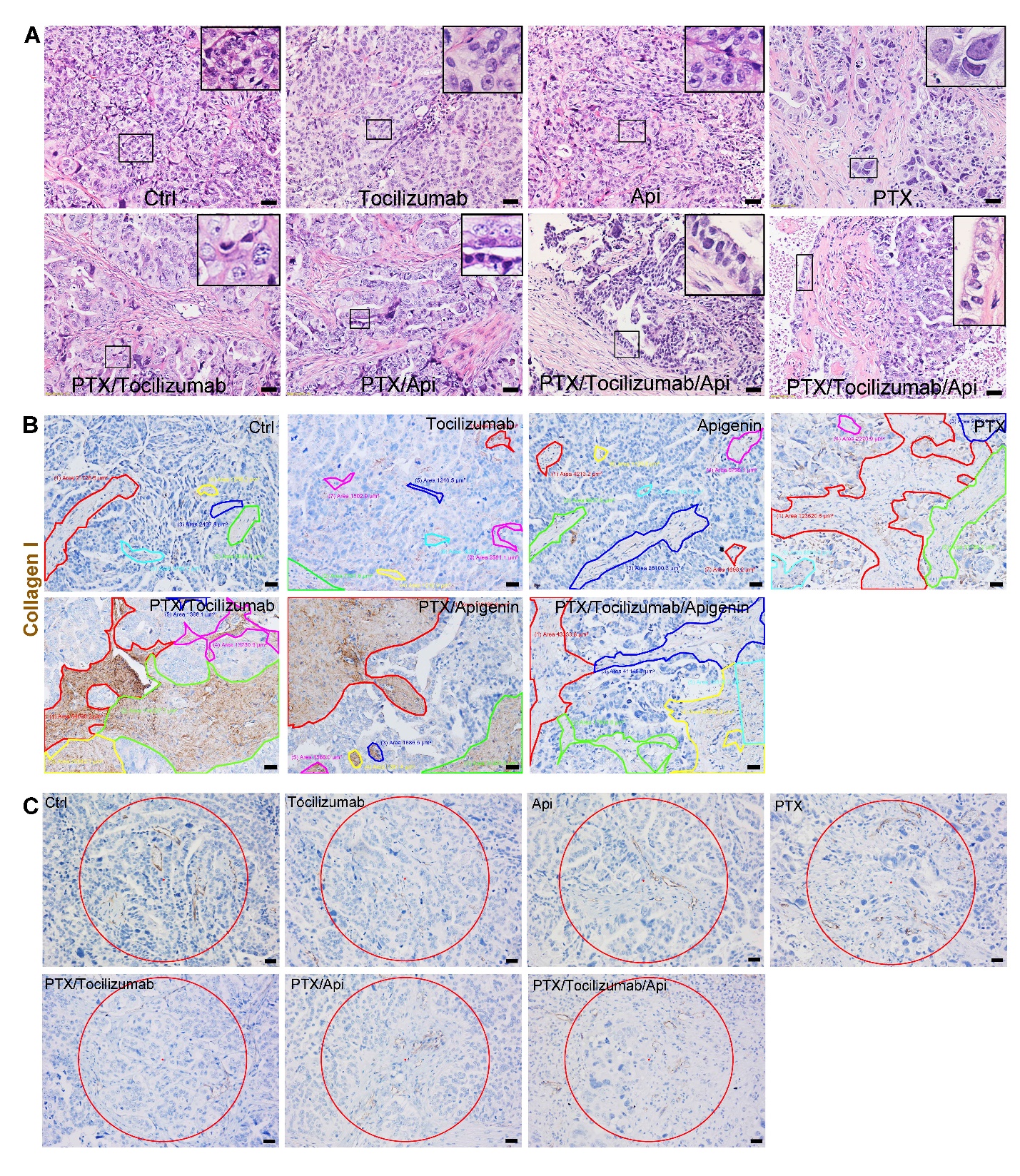


**Supplemental Fig. 4. Collagen deposit and microvessel enrichment in tumor stroma.**

1. Representative photos of histology in test groups, showing the morphology of cancer cells. The panel corners are magnifications of the squared areas.
2. Stroma area was bordered according to the positivity of collagen I. Bars, 50 µm.
3. Microvessel density was demonstrated with CD31 positivity and calculated within a 500-µm-diameter area. Bars, 50 µm.
