# Supplementary material for: IL-6 promotes drug resistance through formation of polyploid giant cancer cells and stromal fibroblast reprogramming
Source: Oncogenesis. 2021 Sep 29;10(9):65. doi: 10.1038/s41389-021-00349-4 (PMC8481288; doi:10.1038/s41389-021-00349-4)
Supplement: Supplementary file 2 — Supplementary Tables [file 41389_2021_349_MOESM2_ESM.docx]

**Supplementary Table 1 Primer Sequence for RT-qPCR Verification**

| **Gene Name** | **Sense** | **Antisense** |
| --- | --- | --- |
| AFP | AGCTTGGTGGTGGATGAAAC | TCTGCAATGACAGCCTCAAG |
| BAX | GAGAGGTCTTTTTCCGAGTG | GG TGAGGAGGCTTGAGGAGT |
| BCL-2 | GCTACCTAAGAAAAACCTGG | CAAGAAACAAGGTCAAAGGG |
| CCL3 | TTCAGAAGGACACGGGCAGCAGACA | GGAATCTGCCGGGAGGTGTAGCT |
| CCL5 | TACATTGCCCGCCCACTGCC | GGGTTGGCACACACTTGGCG |
| CD10 | ACAATGATCGCACTCTATGCAACC | AGGCTCAGTGGTGGCATCCATG |
| CD16 | CACCATCACTCAAGGTTTGG | AGTCCTGTGTCCACTGCAAA |
| CD64 | GCAGGAACACATCCTCTGAA | GTAACTGGAGGCCAAGCACT |
| CSF2 | TGCAGCATCTCTGCACCCGC | AGGCAGGTCGGCTCCTGGAG |
| CXCL1 | AGCCTGCAACATGCCAGCCA | TGTGCACATACATTCCCCTGCCT |
| CXCL10 | TGCAAGCCAATTTTGTCCACGTGT | GCAGCCTCTGTGTGGTCCATCC |
| CXCL3 | AATGTAAGGTCCCCCGGACCCC | ACCACCCTGCAGGAAGTGTCAA |
| CXCL5 | AATCTCCGCTCCTCCACCCAGT | GCTCTCTCAACACAGCAGCGGC |
| DAZL | GCCACGTCCTTTGGTTTTTA | TGGTTGTGGGCTGCATATAA |
| DNMT1 | GGAGAGGCTAAGCGTTCAAG | AAATGAGATGTGATGGTGGTTTG |
| DNMT3A | AAGAGCACAGCGGAGAAG | GCAGATGTCCTCAATGTTCC |
| DNMT3B | CCATGAAGGTTGGCGACAA | TGGCATCAATCATCACTGGATT |
| DNMT3L | GGGACAACTGAAGCATGTGGT | AAGATCGAAGGGTCCCCACT |
| GAPDH | CCCCTCTGCTGATGCCCCCA | TGACCTTGGCCAGGGGTGCT |
| GATA4 | CCTGCGGCCTCTACATGA | AAGGAGCTGCTGGTGTCTTA |
| ICAM4 | AATACACTTTGCGCTGCCACGTG | GGCTCCAAGCGAGCATCAGTGT |
| IGHG1 | GACTGGCTGAATGGCAAGGAG | GGCGATGTCGCTGGGATAGAA |
| IL-1β | AGGCACAAGGCACAACAGGCT | AACAACTGACGCGGCCTGCC |
| IL-6 | CATTCTGCCCTCGAGCCCACC | GGCAGCAGGCAACACCAGGA |
| MMP1 | GTGTCTCACAGCTTCCCAGCGAC | GCACTCCACATCTGGGCTGCTTC |
| MMP3 | TGAGGACACCAGCATGAACC | ACTTCGGGATGCCAGGAAAG |
| MMP9 | GCAAGGGCGTCGTGGTTCC | GGTCGTCGGTGTCGTAGTTGG |
| PAX6 | TGTCCAACGGATGTGTGAGT | TTTCCCAAGCAAAGATGGAC |
| PDL1 | GCCGAAGTCATCTGGACAAG | TCTCAGTGTGCTGGTCACAT |
| SMA | ACTGGGACGACATGGAAAAGA | GGCAACACGAAGCTCATTGTAG |
| TET1 | GGGCACCCTACCGACAGAAGATGC | CTTCTGGGGCTTGGGCTTCTACC |
| TET2 | GGATGTCCTATTGCTAAGTGG | GAATCACAATCACTGCAGCCTC |
| TLR4 | CCCTGCGTGGAGGTGGTTCCTA | CTCCCAGGGCTAAACTCTGGATGGG |
| TNF-α | CCCTCTGGCCCAGGCAGTCA | ATGGGTGGAGGGGCAGCCTT |
| TUBA1C | CCGGGCAGTGTTTGTAGACTTGG | ATCTCCTTGCCAATGGTGTAGTGCC |
| VCAM1 | AGGTGACGAATGAGGGGACCACA | CCAGCCTCCAGAGGGCCACT |
| VEGF | TATTTGACTGCTGTGGACTTGA | GCTGGGTTTGTCGGTGTT |

**Supplementary Table 2 Antibody Details**

| **Antibody** | **RESOURCE** | **Cat no** | **Company** | **Application** |
| --- | --- | --- | --- | --- |
| a-SMA | Mouse | a5228 | SIGMA | WB (43KD, 1:400), IHC (1:200), IF |
| ATM | Rabbit | 2873 | Cell signaling | WB (1:1000) |
| B-actin | Mouse | A-1978 | Sigma | WB (43 kD, 1:5000), |
| CD10 | Rabbit | ab73409 | Abcam | IHC/IF (1:200), Flow (1:500) |
| CD31 | Rabbit | ab28364 | Abcam | IHC (1:200) |
| Chk2 | Mouse | 3440 | Cell signaling | WB (62 kD; 1:1000) |
| Collagen I | Rabbit | ab34710 | Abcam | WB (120 kD, 1:1000), IHC (1:500) |
| Fibronectin | Rabbit | NB110-56989 | Novus | WB (250 kD, 1:1000-10000) |
| GPR77 | Mouse | 342402 | BioLegend | IHC/IF (1:100), Flow (1:250) |
| IL-6 | Rabbit | ab233706 | Abcam | WB (23 kD, 1:1000) |
| IL-6Rα | Mouse | SC-373708 | Santa cruz | WB (80 kD, 1:1000) |
| LOX | Rabbit | ab31238 | Abcam | WB (32 kD; 1:1000) |
| MDR1 | Rabbit | 13978 | Cell signaling | WB (130-180 kD, 1:1000), IHC (1:200) |
| Nanog | Mouse | 4893 | Cell signaling | WB (42 kD, 1:1000), IHC (1:2000) |
| OCT4 | Rabbit | 2750 | Cell signaling | WB (45 kD, 1:1000), IHC (1:200) |
| p44/42-Erk1/2 | Rabbit | 4695 | Cell signaling | WB (42,44 kD; 1:1000), IHC (1:200) |
| P-ATM | Rabbit | 13050 | Cell signaling | WB (1:1000) |
| p-Chk2 | Rabbit | 2197 | Cell signaling | WB (62 kD; 1:1000) |
| P-p44/42-Erk1/2 | Rabbit | 9101 | Cell signaling | WB (42,44 kD; 1:1000) |
| p-stat3 | Rabbit | 9131s | Cell signaling | WB (86 kD, 1:1000) |
| SOX2 | Mouse | mab2018 | R&D | WB (45 kD, 1:500), IHC (1:50) |
| SOX4 | Rabbit | ab86809 | Abcam | WB (52 kD, 1:500-1000), |
| SSEA1 | Mouse | 4744 | Cell signaling | IHC (1:200) |
| stat3 | Rabbit | sc-483 | Santa cruz | WB (86 kD, 1:1000) |
| Vimentin | Mouse | ab8069 | Abcam | WB (57 kD, 1:1000), IHC (1:100) |
